# Cerebral toxoplasmosis in Acquired Immunodeficiency Syndrome (AIDS) patients also provides unifying pathophysiologic hypotheses for Holmes tremor

**DOI:** 10.1186/1471-2377-10-37

**Published:** 2010-06-03

**Authors:** Alain Lekoubou, Rodrigue Njouoguep, Callixte Kuate, André Pascal Kengne

**Affiliations:** 1 Hôpital Neurologique et neurochirurgical Pierre Wertheimer, Lyon, France; 2 Faculty of Medicine and Biomedical Sciences, University of Yaoundé, Yaoundé, Cameroon; 3 Department of Neurology, Central Hospital Yaounde, Faculty of Medicine and Biomedical Sciences, University of Yaoundé, Yaoundé, Cameroon; 4 The George Institute for International Health, The University of Sydney, Australia

## Abstract

**Background:**

Holmes tremor is a rare symptomatic movement disorder. Currently suggested pathophysiological mechanisms of the disease are mostly derived from stroke cases. Although rare, cerebral toxoplasmosis may strengthen the pathophysiologic mechanism of disease.

**Case presentation:**

A case of Holmes tremor secondary to cerebral toxoplasmosis in an AIDS patient is presented. A relevant literature search was performed, using pubmed and several entries for Holmes tremor as labelled in the literature. The unifying feature of our case and those of the literature is the involvement of either the cerebello-thalamo-cortical and/or the dentato-rubro-olivary pathways. The abscess or the extension of surrounding edema beyond these two circuits may account for the superimposed dysfunction of the nigrostriatal system in some but not all cases. The short delay observed in our observation and the dramatic response to treatment may indirectly support the secondary neuronal degeneration theory in the mechanism of Holmes tremor.

**Conclusion:**

Cases of cerebral toxoplasmosis in AIDS patients also provide arguments for the role of the thalamo-cortical and/or the dentato-rubro-olivary pathways dysfunction in the pathogenesis of Holmes tremor. Involvement of the nigro-striatal pathway may not be crucial in the development of this syndrome. Our case also brings additional indirect arguments for the role of secondary neuronal degeneration in the mechanism of Holmes tremor.

## Background

Holmes tremor is a rare symptomatic movement disorder [1]. It has a predominantly proximal distribution in the limbs and is characterized by its large amplitude, low frequency (less than 4 Hz), and postural and action patterns that worsen during movement and markedly increase in goal directed movements [2,3]. We report on a case of Holmes tremor in an acquired immune-deficiency syndrome (AIDS) patient with cerebral toxoplasmosis. Because of the location of the lesion in the postero-lateral thalamus, the extension of surrounding edema to the brain stem and the short delay from initial neurological deficit to tremor onset, this case may strengthen the currently suggested pathophysiological mechanisms of the disease.

## Case presentation

A 35-year old heterosexual man consulted for fatigue and speech disturbances on the 17^th ^January 2009 at the Yaounde University Hospital Center. One week before, he started complaining of head dullness and slurred speech. His weight had dropped by 22%, from 72 to 56 kg over the past few months during which he also had persisting fever.

**Figure 1:**
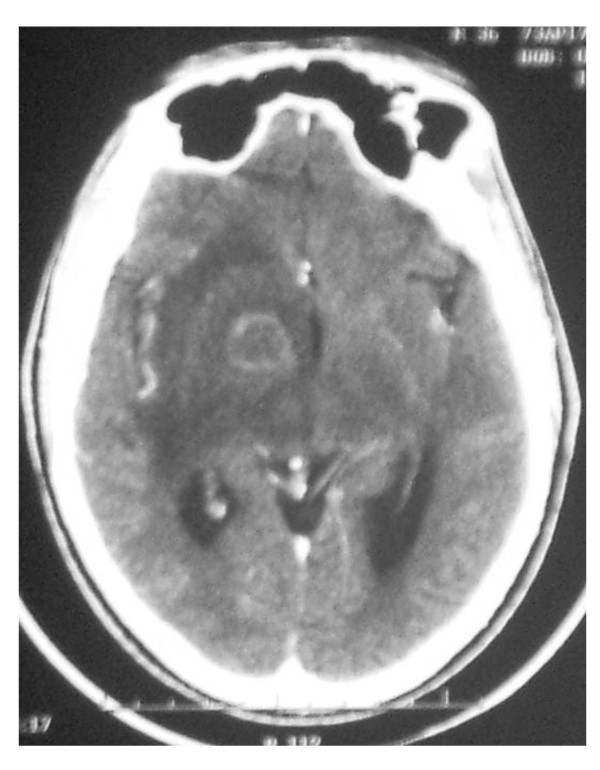
**Brain computerized tomography showing a ring-enhancing lesion in the right thalamus and internal capsule with edema extending downward to the upper mesencephalum**.

On neurological examination, he was confused and had cerebellar dysarthria. His pupils were symmetric with neither ophtalmoplegia nor ptosis. Facial sensation, swallowing and gag reflexes were normal. There was a left-sided hemiparesis with reduced sensation to pain and touch. There was a left Babinski sign. Reflexes were brisk on both sides, and no abnormal movement was present. A brain scan showed a ring-enhanced lesion in the right thalamus with edema extending downward to the upper midbrain (Figure 1). Human Immunodeficiency Virus 1 (HIV-1) serology was positive with a CD4 count of 14 cells/mm3. Viral load was not available. Full blood count showed moderate anemia (Hemoglobin 10.4 g/dl) and lymphopenia (1075 cells/mm3). Serum glutamate pyruvate transaminase and serum glutamate oxalate transaminase were respectively 61 and 62 IU/l. Hepatitis C and Hepatitis B viruses' serologies were negative. Immunoglobulin G anti-toxoplasmic antibodies were positive (1/1012). Sulfadiazine 4 g/pyrimethamine 25 mg daily and Methyl-prednisone 80 mg daily were started 3 days after admission. He was prescribed Lamivudine 30 mg/Zidovudine 300 mg 12 hourly, Efavirenz 600 mg daily and Fluconazole 200 mg daily for cryptococcal meningitis prophylaxis. On day-8 of admission, the patient was less confused, but developed a low frequency tremor of the upper and lower left limbs with jerky-like patterns. It was a postural and intention tremor with a more discreet resting component. No dystonic posture was noted (Additional file 1). No tremor was observed in the paretic limbs. Electroencephalography was normal. Clonazepam 1 mg 12 hourly and trihexyphenidyl 5 mg 12 hourly were added to his treatment, with a reduction in the tremor 24 hours after and a complete resolution 8 days later (Additional file 2). On discharge, 3 weeks after admission, his speech was fluent and motor power was normal on all limbs.

## Discussion

Holmes tremor was originally described by Benedikt and Souquesso and then by Holmes in 1904 who reported a patient with midbrain lesion and a **3-5 **Hz tremor that was present at rest and increased markedly during intentional movement or with certain sustained postures [2,3]. There have been several terms used in the literature to designate this unique tremor. Using the terms "Holmes tremor", "midbrain tremor", "myorhythmia", "thalamic tremor", "mesencephalic tremor" and searching through Medline, we found only 5 cases of Holmes tremor complicating cerebral toxoplasmosis in AIDS patients. Mattos et al reported one case of midbrain toxoplasmosis and one case of midbrain and cerebellar toxoplasmosis [4] while Koppel [5] described a case with midbrain lesion. Strecker and al [6] reported on a case of mesencephalic toxoplasmic abscess involving the red nucleus and extending to the cerebellar peduncle. In the case reported by Pezzini [1], multiple nodular lesions were found in the post-central gyrus, in the right frontal superior gyrus, in the inferior parietal lobule and more importantly in the thalamus extending to the midbrain. There is one report of a patient with Holmes' tremor and toxoplasmic abscess in the left posterior thalamic region and in the posterior arm of the internal capsule [7]. Our case has two specific features: there was an abscess in the thalamus and the internal capsule with extensive edema involving the midbrain and the very short delay from initial neurological deficit to tremor onset. Because in our patients, the postero-lateral thalamus was involved, the cortico-thalamic tracts may have therefore been damaged. In all cases of Holmes tremor in AIDS patients with cerebral toxoplasmic abscess reported in the literature, various anatomical locations of brain abscesses were associated with Holmes tremor (Table 1). The unifying feature of all these observations is the involvement of either the cerebello-thalamo-cortical and/or the dentato-rubro-olivary pathways. Involvement of the same tracts have also been reported in Holmes tremor due to other causes, especially those secondary to vascular or brain traumatic lesions [3]. In our patient, the role of a superimposed dysfunction of the nigrostriatal system either by the abscess in the internal capsule or surrounding edema may account for the rest component, although it remains hypothetical as in the case reported by Micheli [2,3]. The nigrostriatal system was not involved in all reported cases of toxoplasmic-related Holmes tremor. There is one report of DaTSCAN SPECT study in AIDS and Holmes tremor related to a toxoplasmic abscess [6]. The author showed that there was a left-sided reduction of dopamine transporter 4 months following a treatment of cerebral toxoplasmosis. Recently, in a retrospective DaTSCAN SPECT study of six patients with Holmes tremor, there was no remarkable visual difference in presynaptic dopaminergic nigrostriatal system involvement. The authors concluded that nigrostriatal pathway damage may not be crucial for the development of Holmes tremor [8]. Finally, it is also possible that direct HIV- infections of neural cells in these pathways play a modulatory pathophysiologic role thus explaining why not all patients with cerebral toxoplasmosis (which predominantly involves basal ganglia) develop Holmes tremor.

**Table 1:** Cases of Holmes tremor in patients with AIDS and cerebral toxoplasmosis.

First author, Year of publication	Location of brain abscess	Involvement of the cerebello-thalamo-cortical and/or dento-rubro-olivary pathway	Involvement of the nigro-striatal pathway
**Lekoubou A**,	- Right posterior thalamus - Internal capsule	Yes	No/hypothetical

**Strecker K, 2006**	- Left midbrain	Yes	Yes

**Pezzini A, 2002**	- Left frontal superior gyrus, - Inferior parietal lobule, - right thalamus, - right midbrain	Yes	Yes

**Mattos JP, 2002**	- Left midbrain - Left cerebellar hemisphere	Yes	Yes

**Micheli F, 1997**	- Left posterior thalamus - Posterior arm of internal capsule	Yes	No/Hypothetical

**Koppel S, 1990**	- Left midbrain - Left frontal white matter	Yes	Yes

Radio-clinical correlations and possible role of the cerebello-thalamo-cortical and/or dento-rubro-olivary and nigro-striatal pathways.

In our patient, the delay from initial neurological deficit to the onset of tremor was only eighteen days. In previous reports, this delay (when available) ranged from 1 month to 5 months [4-7]. The role of a secondary degeneration in the mechanisms of tremor has been advocated as it usually arises as a delayed manifestation of the initial lesion [3]. In our observation and in that of Mattos [4], tremor occurred within one month of initial neurological deficit. Unlike other cases of Holmes tremor including those related to toxoplasmic abscess, in these two cases, there was a dramatic improvement of the tremor while on antitoxoplasmic/steroid treatment. It is likely that in our case, neuronal integrity was restored before degeneration was initiated and very unlikely that the improvement could have occurred spontaneously as reported cases of spontaneous relief occurred within one year of tremor onset [9].

## Conclusion

Our case further illustrates the role of neural pathways namely the cerebello-thalamo-cortical and/or the dentato-rubro-olivary in the pathogenesis of Holmes tremor. Put together, reported cases of Holmes tremor in the setting of AIDS and toxoplasmic abscess suggest that involvement of the nigro-striatal pathway may not be crucial in the development of this syndrome. Our case also brings additional indirect arguments for the role of secondary neuronal degeneration in the mechanism of Holmes tremor.

## Consent

Written informed consent was obtained from the patient for publication of this case report and any accompanying images/video. A copy of the written consent is available for review by the Editor-in-Chief of this journal.

## Competing interests

Financial disclosure related to research covered in this article: None for all authors

## Authors' contributions

Clinical work-up and literature search were performed by AL, RD and APK. All authors made critical contributions to the paper and approved the final manuscript.

## Pre-publication history

The pre-publication history for this paper can be accessed here:

http://www.biomedcentral.com/1471-2377/10/37/prepub

## Supplementary Material

Additional file 1 **Video file of the tremor at onset**. This file shows the characteristic features of Holmes tremor observed in the patient. Click here for file

Additional file 2 **video file after treatment**. This file shows a complete resolution of the tremor after treatment. Click here for file
